# Detecting lineage-defining mutations in SARS-CoV-2 using colorimetric RT-LAMP without probes or additional primers

**DOI:** 10.1038/s41598-022-15368-3

**Published:** 2022-07-07

**Authors:** Carlos Abelardo dos Santos, Lívia do Carmo Silva, Marcio Neres de Souza Júnior, Geovana de Melo Mendes, Paulo Felipe Neves Estrela, Kézia Gomes de Oliveira, Juliana Santana de Curcio, Paola Cristina Resende, Marilda Mendonça Siqueira, Alex Pauvolid-Corrêa, Gabriela Rodrigues Mendes Duarte, Elisângela de Paula Silveira-Lacerda

**Affiliations:** 1 Laboratório de Genética Molecular e Citogenética, Departamento de Genética, Instituto de Ciências Biológicas I, Universidade Federal de Goiás, Goiânia, Goiás State 74001-970 Brazil; 2 Instituto de Química, Universidade Federal de Goiás, Goiânia, Brazil; 3 Laboratory of Respiratory Viruses and Measles, Reference Laboratory for COVID-19 (WHO) of Oswaldo Cruz Foundation (Fiocruz), Rio de Janeiro, Brazil; 4 Department of Veterinary Integrative Biosciences, Texas A&M University, College Station, TX USA

**Keywords:** Biotechnology, Molecular biology

## Abstract

Despite the advance of vaccination worldwide, epidemic waves caused by more transmissible and immune evasive genetic variants of SARS-CoV-2 have sustained the ongoing pandemic of COVID-19. Monitoring such variants is expensive, as it usually relies on whole-genome sequencing methods. Therefore, it is necessary to develop alternatives that could help identify samples from specific variants. Reverse transcription loop-mediated isothermal amplification is a method that has been increasingly used for nucleic acid amplification, as it is cheaper and easier to perform when compared to other molecular techniques. As a proof of concept that can help distinguish variants, we present an RT-LAMP assay capable of detecting samples carrying a group of mutations that can be related to specific SARS-CoV-2 lineages, here demonstrated for the Variant of Concern Gamma. We tested 60 SARS-CoV-2 RNA samples extracted from swab samples and the reaction showed a sensitivity of 93.33%, a specificity of 88.89% and a kappa value of 0.822 for samples with a Ct ≤ 22.93. The RT-LAMP assay demonstrated to be useful to distinguish VOC Gamma and may be of particular interest as a screening approach for variants in countries with poor sequencing coverage.

## Introduction

Mutations are inevitably introduced in the viral genome during the replication process in host cells. Genetic and evolutionary characteristics such as the large genome and high mutation rate give the coronaviruses a high level of genetic plasticity^1^. In this sense, the Severe Acute Respiratory Syndrome Coronavirus 2 (SARS-CoV-2) has undergone several mutations over time that have resulted in genetic variations, which have sustained the ongoing pandemic^2,3^. Mutations have been defined by mapping changes in the genetic sequence of the SARS-CoV-2 virus compared to Wuhan-Hu1 reference sequence in a given population^4^. In this way, in early 2022, over 1.600 lineages have been suggested at the PANGO Lineages network (https://cov-lineages.org/lineage_list.html updated on 26-02-2022). These introductions rarely settle in the population, and very few can potentially impact the properties of the virus^5^. However, the combination of certain mutations has demonstrated the capacity to alter viral transmissibility, and these variants are called variants of concern (VOCs)^6^. Besides faster spread, VOCs have been related to resistance to antibody neutralization and decreased response to available diagnostics^7^.

Five main VOCs that chronologically upsurged in Europe (Alpha/B.1.1.7/Q.*), Africa (Beta B.1.351/), Americas (Gamma/P.1/P.1.*), Asia (Delta/B.1.617.2/AY.*) and Africa (Omicron/BA.1.1.529/BA.*) spread around the world^8,9^. The Gamma variant first appeared in the state of Amazonas in northern Brazil in December 2020^10^, and by March of 2021 was the most prevalent VOC nationwide being suppressed only in August 2021 by the Delta variant (http://www.genomahcov.fiocruz.br/dashboard-en/). In December 2021, Omicron rapidly overtook all circulating variants in Brazil^11^.

Monitoring emerging variants using genetic sequencing techniques is essential for guiding science-based public health policies. In this sense, with 11.1% of all positive cases sequenced and deposited to the EpiCoV database in GISAID, the United Kingdom is one of the countries with larger sequencing coverage. On the other hand, Brazil, which has the third-highest number of confirmed cases in the world, has sequenced only about one (0.4%) in every 243 positive cases (https://www.gisaid.org/submission-tracker-global/). This situation can be even more challenging in other developing countries, where the lack of funding and structure has resulted in a severe blackout of genomic data, as observed in Bolivia, Kyrgyzstan and Honduras, which have deposited the sequences of about 0.05% of all positive cases^12,13^.

Although whole-genome sequencing is the gold standard for identifying and detecting SARS-CoV-2 variants, some studies have proposed real-time quantitative reverse transcription polymerase chain reaction (RT-qPCR) with variant-specific primers and probes to increase surveillance capacity, with lower execution costs^14–17^. These assays target lineage-defining mutations and can indirectly detect some of the variants that are currently circulating^18^. This approach has also been used to detect VOCs in matrices that SARS-CoV-2 has been less successfully sequenced, such as wastewater^19^.

Although the use of RT-qPCR in lineage-defining assays has represented an advance in SARS-CoV-2 surveillance, the method still has cost-related limitations in countries with inadequate laboratory infrastructure. Other techniques, such as Reverse Transcriptase Loop-Mediated Isothermal Amplification (RT-LAMP), can potentially simplify the execution of the test. The RT-LAMP has been proposed as a reliable diagnostic tool for detecting SARS-CoV-2^20–23^. This method can detect the target genome with high specificity and sensibility in < 40 min, without the need for a real-time thermocycler. However, regular RT-LAMP reactions do not have enough specificity to detect point mutations^24^. Thus, several modifications to the technique have been proposed to address this issue^25,26^. These modifications that increase RT-LAMP specificity can be broadly divided into primer-based and probe-based approaches^24^. In general, these approaches use additional primers or primers and probes to accelerate the amplification of targets harbouring the desired single nucleotide polymorphisms (SNPs) whilst delaying the amplification of the targets that do not contain them.

In this study, we develop a set of primers for RT-LAMP capable of detecting the specific mutations C21614T (also known as S: L18F) and C21638T (also known as S: P26S) that are lineage-defining mutations to the VOC Gamma and descendent lineages. Although these mutations might also be found in the Beta variant, according to data extracted from GISAID, only 11 cases were reported in Brazil. The frequency in which these two mutations can be found in other variants of concern is displayed in Fig. S1. The proposed RT-LAMP assay reported here was able to discriminate samples containing Gamma-specific mutations, indirectly monitoring its prevalence. To our knowledge, this is the first report of RT-LAMP being used to detect mutations to detect the VOC Gamma of SARS-CoV-2 without probes or additional primers. The current test serves as a proof of concept that RT-LAMP has the potential to aid in increasing variant surveillance, as it can differentiate variants without the need for expensive molecular beacons.

## Results

### Optimization of the RT-LAMP assay

To assess the optimal temperature, we performed RT-LAMP at temperatures ranging from 60 to 70 °C. Additionally, samples were evaluated at different times from 20 to 120 min. The best temperature for variant-specific amplification was 65 °C and the time required was 70 min. For time points longer than 70 min, some negative reactions would become orangish and yellowish. This colour change was probably caused by spurious amplification, a well-documented problem with RT-LAMP reactions^27^.

**Figure 1:**
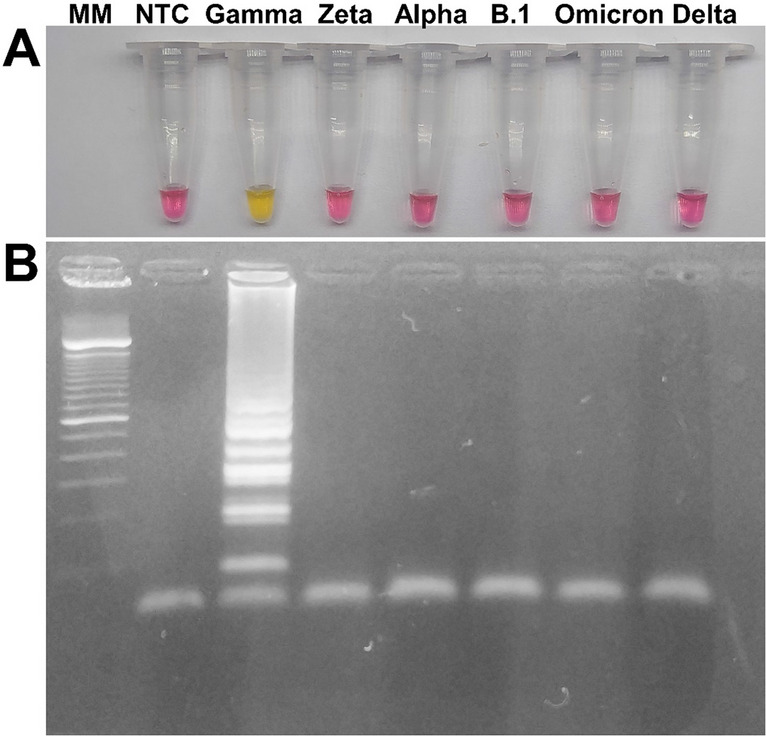
Specificity of the RT-LAMP reaction incubated at 65 °C for 70 min. **A**: RT-LAMP amplification of the Gamma variant, among RNA samples from different lineages of SARS-CoV-2 as a target. The WarmStart Colorimetric Kit uses a pH indicator that turns from pink to yellow in the presence of amplicons. **B**: Visualisation of amplicons using 2% agarose gel electrophoresis. The positive sample presents the typical ladder-like pattern common to RT-LAMP fragments. MM: Molecular Marker, NTC: Non-template control using H_2_O instead of a target.

In the optimized temperature and time, cross-reactivity was evaluated against the Gamma, Zeta, Alpha, B.1, Delta, and Omicron variants. As shown in Fig. 1, only in the presence of the Gamma variant the reaction showed positivity, both through colorimetric detection and by agarose gel.

### Analytical sensitivity

**Figure 2:**
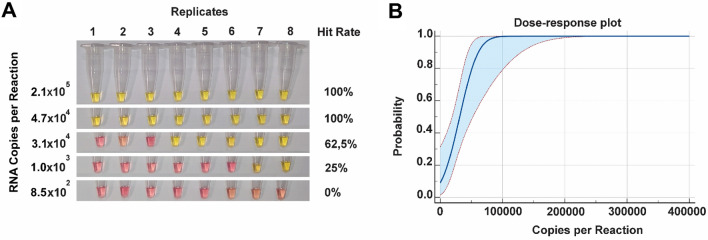
Determination of the detection limit of the LAMP assay by Probit analysis using different analyte concentrations with a total of eight replicates per concentration. (**A**) Eight independent replicates per dilution were performed. The number of RNA copies per reaction is displayed on the left, and the Hit Rate (%) is displayed on the right (**B**). Probit regression analysis with a sigmoid dose–response curve of the concentration–response data.

The Limit of Detection (LoD) of the test is the minimum number of RNA copies that can be detected by the method 95% of the time. When assaying samples carrying a number of copies near the detection limit, the amplification is stochastic, i.e., may or may not occur. To correctly estimate the LoD, we diluted the RNA from 2.1 × 10^6^ to 8.5 × 10^2^ copies per reaction and quantified each point by RT-qPCR. We tested eight replicates of each point using RT-LAMP and performed a probit analysis. Given a C_95_, the limit of detection according to the probit analysis is 4.8 × 10^4^ copies per reaction which corresponds to a Ct of ≅ 22.93. The results are shown in Fig. 2.

### Validation in clinical samples

To assess whether this RT-LAMP assay could be used to differentiate samples carrying the lineage-defining mutations in field samples, we included RNA samples from 60 swabs confirmed positive for SARS-CoV-2 by RT-qPCR and sequencing. GISAID and/or GenBank accession codes are displayed in Table S1. The reactions were performed in triplicates, and sequencing data was only retrieved after the RT-LAMP results had been analysed. Samples were pre-selected based on Ct, and only samples with a medium Ct for the N1 and N2 primer sets ≤ 22.93 were included. We used 1.5 μL of target RNA in a 15 μL reaction. Table 1 shows the Ct values as quantified by the RT-qPCR, the sequencing results as categorized by the Pangolin lineage assigner (https://cov-lineages.org/resources/pangolin.html) and the results for the three RT-LAMP replicates.

**Table 1:** Comparison between sequencing and RT-LAMP results for field samples.

Sample^1^	Ct^2^	Sequencing Results^3^	RT-LAMP Results^4^		
		Variant	Replicate 1	Replicate 2	Replicate 3
B25	18.17	Alpha	+	−	−
B29	16.43	Alpha	−	−	−
B48	20.55	Alpha	−	−	−
B186	20.47	Zeta	−	−	−
B195	19.19	B.1.1	−	−	−
B211	19.58	Zeta	−	−	−
B212	16.32	Gamma	+	+	+
B213	15.07	Gamma	+	+	+
B214	14.46	P.1.12	+	+	+
B215	14.91	Gamma	+	+	+
B216	16.38	P.1.7	+	+	+
B217	14.62	Gamma	+	+	+
B218	12.03	Gamma	+	+	+
B219	22.30	Gamma	+	+	+
B220	14.22	Gamma	+	+	+
B221	14.46	Gamma	+	+	+
B222	21.53	Gamma	−	+	−
B223	18.61	Gamma	+	+	+
B224	18.63	Gamma	+	+	+
B225	20.94	Gamma	−	+	−
B227	15.92	Gamma	+	+	+
B228	22.38	Gamma	−	+	−
B229	19.36	Gamma	+	+	+
B230	18.82	Gamma	+	+	+
B231	18.49	Gamma	+	+	+
B236	21.89	Zeta	−	−	−
B240	18.13	Zeta	−	−	−
B244	20.28	P.7	−	−	−
B245	16.80	P.7	−	−	−
B247	17.17	Gamma	+	+	+
F1	12.89	Gamma	+	+	+
F2	17.51	P.1.7	+	+	+
F3	13.89	Gamma	+	+	+
F4	19.44	P.1.7	+	+	+
F5	17.20	P.1.7	+	+	+
F6	16.30	Gamma	+	+	+
F7	21.98	P.1.7	+	+	+
F8	20.48	P.1.7	+	+	+
F9	21.26	P.1.7	+	+	+
F10	15.92	Gamma	+	+	+
F11	21.97	Delta	−	−	−
F12	21.07	Delta	−	−	−
F13	20.61	Delta	−	−	−
F14	19.29	Delta	−	−	−
F15	18.11	Delta	−	−	+
F16	17.77	Delta	−	−	+
F17	16.01	Delta	+	+	+
F18	15.09	Delta	+	+	+
F19	15.96	Delta	+	−	−
F20	21.31	Delta	−	−	−
F21	22.55	Omicron	−	−	−
F22	21.27	Omicron	−	−	−
F23	20.20	Omicron	−	−	−
F24	19.13	Omicron	−	−	−
F25	17.41	Omicron	−	−	−
F26	21.96	Omicron	−	−	−
F27	20.76	Omicron	−	−	−
F28	19.89	Omicron	−	−	−
F29	18.51	Omicron	−	−	−
F30	15.44	Omicron	−	−	−

The cycle threshold of RT-qPCR was used as a sample selection criterion.

*Ct* Cycle threshold.

(−) indicates a negative result (no color change).

(+) indicates a positive result (color change).

^1^Sample identification code based in the list provided by LGBIO laboratory, UFG (B- samples) or FIOCRUZ (F- samples);

^2^The Ct column corresponds to the quantification cycle of the sample positive for SARS-CoV-2 using the 2019nCoV primer kit;

^3^Results of sequencing obtained in the GISAID EpiCoV platform;

^4^Results of RT-LAMP assay in triplicate;

The RT-LAMP assay targeted two mutations of Gamma variant and descendant lineages (P.1.7 and P.1.12). All sequences were aligned using MAFFT Software (https://mafft.cbrc.jp/alignment/server/) and individually checked for the presence of the mutations using BioEdit Software (version 7.2), as shown in Fig. S2. We confirmed that only Gamma and descendant lineages presented the mutations. All data considered for statistical analysis is shown (Table 2). Statistical analysis was performed for each triplicate. The mean specificity of the test was 88.89%, and the sensitivity for samples with a Ct ≤ 22.93 was 93.33%. The mean kappa value was 0.822. The positivity of the samples was assayed by naked eye and confirmed by agarose gel electrophoresis, as can be seen in Fig. S3. When loaded to the agarose gel, positive samples present the typical LAMP pattern. It is possible to visualize a distinct banding pattern on the agarose gel for sample code B48 in A, samples codes F16, F23 and F25 in D, samples B240 and B245 in F, F16, F23 and F28 in H, samples B29 and B211 in I, sample B228 in J and F13 in K, which are probably the result of spurious amplification. Since there was no color change to yellow, these samples were considered negative.

**Table 2:** Summary of the RT-LAMP and Sequencing results.

		Sequencing			
		Replicate #	Pos	Neg	Sum
RT-LAMP	Pos	#1	27	4	31
		#2	30	2	32
		#3	27	4	31
	Neg	#1	3	26	29
		#2	0	28	28
		#3	3	26	29
		Sum	90	90	180

All Gamma and descendant lineages were confirmed positive by sequencing. Even though positive for SARS-CoV-2, samples that did not have the target mutations were marked as “negative” in this table. Rows show the RT-LAMP results and columns show sequencing results. The number in the table is the number of samples in which the results agree or disagree between the two methodologies for each replicate.

*RT-LAMP* Reverse-Transcriptase Loop-Mediated Amplification; *Rep* Replicates; *Pos* Positive; *Neg* Negative.

## Discussion

It is essential to understand how SARS-CoV-2 changes by accumulating mutations during the pandemic. Variants that carry mutations that offer some advantage tend to become established in the population. Some SARS-CoV-2 variants, such as the Gamma variant, display mutations that increase viral transmissibility and result in antibody evasion^28^. The VOCs have been related to the successive epidemic waves worldwide^29^. In this sense, monitoring new variants with greater transmission potential is instrumental for SARS-CoV-2 surveillance. Whole-genome sequencing has allowed the monitoring and detection of novel variants. The presence of these lineage-defining mutations serves as a "signature" that can be also used to identify the variant by other means rather than sequencing.

Recently, several studies have related the RT-LAMP technique to variants of the new coronavirus^30,31^. These papers have focused on developing protocols that allow the detection of SARS-CoV-2, regardless of the variant. Although these works are essential for molecular diagnostic purposes, they cannot be used for epidemiological research to investigate the course of the health crisis, as they do not distinguish the variants. In contrast, RT-LAMP has been used to detect SARS-CoV-2 genomes containing the S1Δ69-70 deletion present in the alpha variant (B.1.1.7)^32^. The authors used molecular beacons and additional oligonucleotides to block nucleic acids. Another study reported an RT-LAMP-based genotyping method focusing on R203M in the SARS-CoV-2 N gene to detect Delta variants specifically^33^. Although the test did not use additional oligonucleotides, the excellent ability to distinguish between Delta and non-Delta variants occurred by calculating a ratio between the Cts of two different reactions per sample. Our study advanced the frontier of knowledge by proposing a new specific method for gamma variants and descendants that contain the mutations C21614T (also known as S:L18F) and C21638T (also known as S: P26S). Our methodology doesn’t rely on the use of additional probes and is independent of the sophisticated instrumentation necessary for protocols that perform conventional qLAMP.

In this study, we designed RT-LAMP primers capable of differentiating samples carrying lineage-defining mutations of the VOC Gamma and its descendant lineages. These mutations can be used as a proxy to identify Gamma with a 91.11% accuracy for samples with Ct ≤ 22.93.

The detection limit of 4.8 × 10^4^ copies (Ct ~ 22.93) found in the present study is higher than those reported in the literature^24–27,34^. Nonetheless, it has excellent potential for the confirmatory diagnosis of the VOC Gamma. The RT-LAMP test is not sensitive enough to detect the virus in patients with a lower viral load. However, it is sensitive enough to detect patients who are in the acute phase of infection (which includes up to the fifth day of infection) in which the load viral load is averaging 6.76 × 10^5^ copies, as demonstrated by Wölfel et al.^35^ in a study performed with samples collected during the clinical course of COVID-19. Considering that this value is substantially higher than the detection limit obtained in this study (4.8 × 10^4^ copies), the proposed RT-LAMP assay here could indirectly monitor the prevalence of Gamma-specific mutations in patients who are within the first days of infection.

The RT-LAMP assay for monitoring emerging variants has shown promising results by providing a sensitivity that is adequate to enter the same workflow as the regular sequencing technique, which is considered the gold standard. In our study, the RT-LAMP method was able to differentiate samples with Ct up to ≤ 22.93. Samples with lower Ct are preferred for sequencing as they correlate with higher coverages, thus allowing better identification of samples^36^. Therefore, the RT-LAMP presented in this study could be used as a screening method in places with a low sequencing capacity.

Another relevant aspect of the RT-LAMP as an alternative to identify variants is the time and cost for test execution compared to other technologies (Table 3). The cost for application of RT-LAMP per sample is 50 and 4.6 times cheaper than Next-generation sequencing (NGS) and RT-qPCR respectively.

**Table 3:** Comparison between the cost, time and principle of different strategies for detection of SARS-CoV-2 variants.

Method	Working principle	Time required	Cost per sample
NGS	The genomic strand is fragmented and the bases of each fragment are identified by signals emitted when the fragments are linked to a template strand	1–2 days	**~ $**150
RT-qPCR	Synthesis of DNA from deoxynucleotide substrates on a single-stranded DNA template by temperature cycling	About 2 h	~ $14
RT-LAMP	Synthesis of DNA from deoxynucleotide substrates on a single-stranded DNA template using a constant temperature	30 min–1 h	~ $3

Another valuable feature of RT-LAMP is the run time that can vary from 30 min to 1 h. The RT-LAMP is a faster method when compared to RT-qPCR which takes at least two hours, and NGS that can take from one to two days. Therefore, RT-LAMP has the potential to be applied as a rapid diagnostic method and valuable tool for the surveillance of VOCs.

Even though it might not be possible to design primers targeting two or more mutations for a great variety of targets, this approach was successful to differentiate samples carrying both mutations in the Gamma variant, as it resulted in different times-to-threshold between the samples that harbored or not the mutations. The time-to-threshold is the amount of time it takes a positive sample to turn from pink to yellow and it is an indicator of how efficiently the amplification occurred. Low viral loads and non-specific amplifications usually take longer to amplify than high viral load specific amplifications, so it is possible to estimate a time with optimal specificity and sensibility ratios. Even though this test is not as sensitive as RT-qPCR, samples infected with the Gamma variant have been found to have a tenfold increase in viral load when compared to non-Gamma samples^10^. This could lower the negative impact of the low sensitivity of the test, as the median Ct for samples infected with Gamma variant is around 19.8^10^.

As vaccination continues worldwide, monitoring emerging variants is vital for guiding public policies and avoiding outbreaks of new variants that might significantly escape antibodies or promote high rates of contamination, as seen recently by the Omicron variant. Gamma variant was the most prevalent variant in Brazil from February 2021 to July 2021, when it was suppressed by the Delta variant^11^. In the present study, we highlight how a lineage-specific RT-LAMP can be a useful tool to estimate the prevalence of variants in the population in a given epidemiological week. An updated set of primers capable of detecting specific mutations of potential novel variants that may surge could be used as a screening method before sequencing, in places where sequencing reagents are expensive or that lack the infrastructure to sequence many samples.

## Methods

### RNA control and biological samples

The Laboratory of Respiratory Viruses and Measles, Reference Laboratory for COVID-19 (WHO) of Oswaldo Cruz Foundation (FIOCRUZ) provided the RNA samples of different SARS-CoV-2 variants (Alpha, Gamma, B.1, Zeta, Delta and Omicron), which we used to optimize the test. During the validation step, we used RNA extracted from swab samples from SARS-CoV-2 positive patients. Samples B25-B247 were kindly provided by the Laboratory of Genetics and Biodiversity (LGBIO) from the University of Goiás (UFG). They performed the SARS-CoV-2 gene sequencing study in accordance with the ethics committee no. 4.365.579. All other samples were provided by the Laboratory of Respiratory Viruses and Measles. Sequence data of the samples were deposited to the EpiCoV database in the GISAID and/or GenBank platforms, and all accession numbers are displayed in Table S1. The samples provided by LBGIO were randomly selected for the RT-LAMP assay, and then tested based on Ct. Sequencing results were only retrieved after the RT-LAMP results had been analyzed. The RNA and biological samples were stored at − 80 °C in the Molecular Genetics and Cytogenetics Laboratory at UFG until use.

### RNA extraction and RT-qPCR

According to the manufacturer's instructions, RNA from the swab samples was extracted using the Viral DNA/RNA Extraction Kit (Cellco, São Paulo, Brazil). The primer and probes used for the detection of the genes of SARS-CoV-2 and human RNase P were the 2019nCoV Kit (Integrated DNA Technologies, Iowa, USA), and the RT-qPCR was performed using the GoTaq probe 1-step RT-qPCR system (Promega, Wisconsin, USA) in an AriaMx Real-Time PCR System (Agilent, California, USA). The amplification program consisted of one cycle at 45 °C for 15 min for reverse transcription, one cycle at 95 °C for 2 min for reverse transcriptase denaturation and DNA polymerase activation, followed by 40 cycles at 95 °C for 3 s, and 55 °C for 30 s for denaturation and amplification.

### RT-LAMP primer design

**Figure 3:**
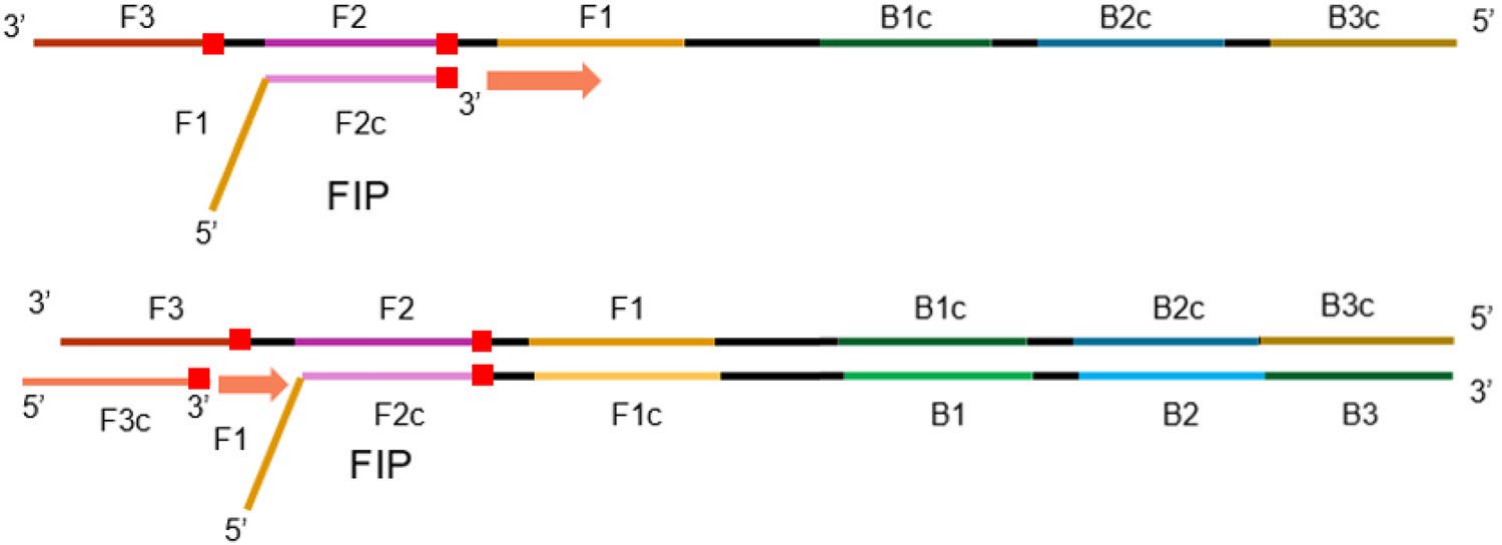
Primers targeting the lineage-defining mutations in the target. The position of the lineage defining mutations within the F3 and F2 primer regions are marked as red squares. FIP = Forward Inner Primer, F3c = Primer complementary to region F3.

The RT-LAMP primer set was designed targeting two lineage-defining mutations in the Gamma variant using Primer Explorer (V.5.0) (available at http://primerexplorer.jp/lampv5e/index.html), according to the developer's instructions. In brief, primers were designed so that the mutations of interest would be located either in the 5' end of the F1c or B1c regions or in the 3' end of F2, B2, F3, or B3 regions. RT-LAMP alone cannot usually differentiate SNPs^18^. To address this issue, we first looked for regions in the genome that would have more than one lineage-defining mutation (Fig. 3). Primers were designed so that the primers that bind to F3 and F2 regions would have the mutation located on its 3’ prime end. In this way, primers would have more affinity binding to mutated targets than wild ones. The use of two primers targeting two different SNPs allowed a certain level of redundancy, being crucial for sample differentiation. This primer set was designed targeting a region in the S gene harboring two mutations (C21614T and C21638T) predominantly present in the Gamma variant (Fig. S4).

The lineage-defining mutations were retrieved from PANGO lineages (https://cov-lineages.org/) and compared against the reference sequence (NC_045512). Primer sequences are displayed in Table 4.

**Table 4:** Nucleotide sequence of RT-LAMP primers designed for this study.

Primer Name	Sequence (5' → 3')	Target	Variant detected
F3	TCTCTAGTCAGTGTGTTAAT**T**	C21614T	Gamma
B3	GACAGGGTTATCAAACCTCT		
FIP	AGGATCTGAAAACTTTGTCAGGGTTTTTTTCAAACAGAACTCAATTACCC**T**	C21638T	
BIP	ACTCAGGACTTGTTCTTACCTTTCTTTTTTTTAGTACCATTGGTCCCAGA		
LF	CACGTGTGAAAGAATTAGTGTAT		
LB	TCCAATGTTACTTGGTTCCATGC		

*F3* Forward Outer Primer; *B3* Backward Outer Primer; *FIP* Forward Inner Primer; *BIP* Backward Inner Primer; *LF* Loop Forward Primer; *LB* Loop Backward Primer. Underlined bold nucleotides highlight the mutation position in the primer.

### RT-LAMP colorimetric assay

We performed the RT-LAMP assay using the WarmStart Colorimetric LAMP 2X Master Mix (New England Biolabs, Hitchin, UK) as described previously^31^. In brief, the final reaction volume was 15 μL consisting of 7.5 μL LAMP master mix, 0.2 µM of each outer primer (F3 and B3), 1.6 µM of each inner primer (FIP and BIP), 0.8 µM of each loop primer (LF and LB), 4.5 μL nuclease-free water (Sigma Aldrich, Missouri, EUA), and 1.5 μL of the RNA sample. The reaction was incubated at 65 °C for 70 min in a ProFlex 3 × 32-well PCR System (Thermofisher, Massachusetts, USA). The WarmStart Colorimetric Assay uses phenol red as an indicator of target amplification, and the color of the reaction changes from pink to yellow in the presence of amplicons (Silva et al.^34^). After amplification, we analyzed the color of the tube visually and confirmed it by agarose gel electrophoresis. For naked-eye visualization, the tubes were aligned over a white sheet of paper, and photos were acquired with a regular mobile phone camera (48MP, Xiaomi Redmi Note 8) with the aid of a rechargeable white LED light. Samples were then loaded into 10µL wells in an agarose gel and analyzed through electrophoresis. The gel was run for 45 min at 100 V and photos of the gel were acquired using a gel documentation system.

### Analytical sensitivity

The RNA control was serially diluted in nuclease-free water and analyzed by RT-qPCR to obtain the corresponding amplification cycle threshold (Ct). The viral genomic copies were calculated by plotting Ct values onto the standard curve constructed based on the 2019-nCoV_N_Positive Control (Integrated DNA Technologies, Iowa, USA). Samples with a concentration ranging from 2.1 × 10^5^ to 8.5 × 10^2^ were analyzed by RT-LAMP in octuplicates. We performed a Probit analysis to determine the limit of detection (LOD) using MedCalc software (Version 19.6.4, MedCalc Software, Ostend, Belgium), giving a value of C_95_ (95% detection probability across all replicates). The RT-LAMP products of amplification were determined by visual observation and confirmed by gel electrophoresis (2% agarose and 0.5% Tris–EDTA–borate (TEB) buffer).

### Optimization of the test

RNA was extracted from the supernatant of cultures of specific variants or from samples confirmed by sequencing to evaluate if primers were annealing specifically to samples containing the targeted mutations. We tested RNA from variants B.1, Zeta, Alfa, Gamma, Delta and Omicron. Results were analyzed visually and by gel electrophoresis (2% agarose and 0.5% Tris–EDTA–borate (TEB) buffer).

### RT-LAMP validation in clinical samples

We selected the RNA from 60 swab samples positive for SARS-CoV-2 that had already been sequenced and quantified by RT-qPCR to validate the performance of RT-LAMP in detecting mutations in clinical samples. Considering the C95 for the test, we pre-selected samples based on Ct and only the 60 samples with Ct ≤ 22.93 were included. The diagnostic test parameters such as sensitivity, specificity, positive predictive value, negative predictive value and accuracy for each triplicate were calculated using MedCalc's Diagnostic Test Evaluation online tool Calculator (available at: https://www.medcalc.org/calc/diagnostic_test.php). After interpreting RT-LAMP results, sequence data were retrieved from GISAID and analyzed using the Pangolin Lineage Assigner (available at: https://pangolin.cog-uk.io/). Sequences were then aligned using MAFFT Software (https://mafft.cbrc.jp/alignment/server/), and we used the BioEdit Software (version 7.2) to confirm the presence of the targeted mutations in all samples tested by RT-LAMP.

## Supplementary Information

Supplementary Information.

## Data Availability

The datasets analysed during the current study are available in the GISAID repository, at www.gisaid.org, and in the GenBank repository, at https://www.ncbi.nlm.nih.gov/genbank/. All accession numbers for samples are displayed in Table S1 in the supplementary file.

## References

[1] Woo PCY, Lau SKP, Yip CCY, Huang Y, Yuen KY (2009). More and more coronaviruses: Human coronavirus HKU1. Viruses.

[2] Gupta RK (2021). Will SARS-CoV-2 variants of concern affect the promise of vaccines?. Nat. Rev. Immunol..

[3] Otto SP (2021). The origins and potential future of SARS-CoV-2 variants of concern in the evolving COVID-19 pandemic. Curr. Biol..

[4] Timmers LFSM (2021). SARS-CoV-2 mutations in Brazil: From genomics to putative clinical conditions. Sci. Rep..

[5] Grubaugh ND, Petrone ME, Holmes EC (2020). We shouldn’t worry when a virus mutates during disease outbreaks. Nat. Microbiol..

[6] Konings F (2021). SARS-CoV-2 Variants of Interest and Concern naming scheme conducive for global discourse. Nat. Microbiol..

[7] Saito A (2022). Enhanced fusogenicity and pathogenicity of SARS-CoV-2 Delta P681R mutation. Nature.

[8] Sanyaolu A (2021). The emerging SARS-CoV-2 variants of concern. Ther. Adv. Infect. Dis..

[9] Viana R (2022). Rapid epidemic expansion of the SARS-CoV-2 Omicron variant in southern Africa. Nature.

[10] Naveca FG (2021). COVID-19 in Amazonas, Brazil, was driven by the persistence of endemic lineages and P.1 emergence. Nat. Med..

[11] Fundação Oswaldo Cruz (FIOCRUZ). SARS-CoV-2 Genomic Surveillance in Brazil. http://www.genomahcov.fiocruz.br/dashboard-en/ (2022).

[12] Adepoju P (2021). Challenges of SARS-CoV-2 genomic surveillance in Africa. Lancet Microbe.

[13] Lancet T (2021). Genomic sequencing in pandemics. The Lancet.

[14] Vogels CBF (2021). Multiplex qPCR discriminates variants of concern to enhance global surveillance of SARS-CoV-2. PLoS Biol..

[15] Wang H (2021). Multiplex sars-cov-2 genotyping reverse transcriptase pcr for population-level variant screening and epidemiologic surveillance. J. Clin. Microbiol..

[16] Vega-Magaña N (2021). RT-qPCR Assays for Rapid Detection of the N501Y, 69–70del, K417N, and E484K SARS-CoV-2 Mutations: A Screening Strategy to Identify Variants With Clinical Impact. Front. Cell. Infect. Microbiol..

[17] Korukluoglu G (2021). 40 minutes RT-qPCR assay for screening Spike N501Y and HV69–70del mutations. bioRxiv.

[18] Adamoski D (2021). Large-scale screening of asymptomatic persons for SARS-CoV-2 variants of concern and Gamma Takeover, Brazil. Emerg. Infect. Dis..

[19] Wurtzer S (2022). SARS-CoV-2 genome quantification in wastewaters at regional and city scale allows precise monitoring of the whole outbreaks dynamics and variants spreading in the population. Sci. Total Environ..

[20] Dao Thi VL (2020). A colorimetric RT-LAMP assay and LAMP-sequencing for detecting SARS-CoV-2 RNA in clinical samples. Sci. Transl. Med..

[21] García-Bernalt Diego J (2021). A simple, affordable, rapid, stabilized, colorimetric, versatile RT-LAMP assay to detect SARS-CoV-2. Diagnostics.

[22] Nawattanapaiboon K (2021). Colorimetric reverse transcription loop-mediated isothermal amplification (RT-LAMP) as a visual diagnostic platform for the detection of the emerging coronavirus SARS-CoV-2. Analyst.

[23] dos Santos C (2021). Detection of SARS-CoV-2 in saliva by RT-LAMP during a screening of workers in Brazil, including pre-symptomatic carriers. J. Braz. Chem. Soc..

[24] Varona M, Anderson JL (2021). Advances in mutation detection using loop-mediated isothermal amplification. ACS Omega.

[25] Badolo A (2015). Detection of G119S ace-1 R mutation in field-collected Anopheles gambiae mosquitoes using allele-specific loop-mediated isothermal amplification (AS-LAMP) method. Malar. J..

[26] Ayukawa Y, Hanyuda S, Fujita N, Komatsu K, Arie T (2017). Novel loop-mediated isothermal amplification (LAMP) assay with a universal QProbe can detect SNPs determining races in plant pathogenic fungi. Sci. Rep..

[27] Gadkar VJ, Goldfarb DM, Gantt S, Tilley PAG (2018). Real-time detection and monitoring of loop mediated amplification (LAMP) reaction using self-quenching and de-quenching fluorogenic probes. Sci. Rep..

[28] Dejnirattisai W (2021). Antibody evasion by the P.1 strain of SARS-CoV-2. Cell.

[29] Sabino EC (2021). Resurgence of COVID-19 in Manaus, Brazil, despite high seroprevalence. The Lancet.

[30] Luo Z (2022). Optimization of loop-mediated isothermal amplification (LAMP) assay for robust visualization in SARS-CoV-2 and emerging variants diagnosis. Chem. Eng. Sci..

[31] Alves PA (2021). Optimization and Clinical validation of colorimetric reverse transcription loop-mediated isothermal amplification, a fast, highly sensitive and specific COVID-19 molecular diagnostic tool that is robust to detect SARS-CoV-2 variants of concern. Front. Microbiol..

[32] Sherrill-Mix S, van Duyne GD, Bushman FD (2021). Molecular beacons allow specific RT-LAMP detection of B1.1.7 variant SARS-CoV-2. J. Biomol. Tech. JBT.

[33] Yang J (2022). RT-LAMP assay for rapid detection of the R203M mutation in SARS-CoV-2 Delta variant. Emerg. Microbes Infect..

[34] Silva LDC (2021). Can a field molecular diagnosis be accurate? A performance evaluation of colorimetric RT-LAMP for the detection of SARS-CoV-2 in a hospital setting. Anal. Methods.

[35] Wölfel R (2020). Virological assessment of hospitalized patients with COVID-2019. Nature.

[36] Goswami C (2022). Identification of SARS-CoV-2 variants using viral sequencing for the Centers for Disease Control and Prevention genomic surveillance program. BMC Infect. Dis..

