# Supplementary material for: Detecting lineage-defining mutations in SARS-CoV-2 using colorimetric RT-LAMP without probes or additional primers
Source: Sci Rep. 2022 Jul 7;12:11500. doi: 10.1038/s41598-022-15368-3 (PMC9261132; doi:10.1038/s41598-022-15368-3)
Supplement: Supplementary file 1 — Supplementary Information. [file 41598_2022_15368_MOESM1_ESM.pdf]

Detecting lineage-defining mutations in SARS-CoV-2 using colorimetric RT-LAMP without probes or additional primers

Carlos Abelardo dos Santos[1], Lívia do Carmo Silva[1], Marcio Neres de Souza Júnior[2], Geovana de Melo Mendes[2], Paulo Felipe Neves Estrela[2], Kézia Gomes de Oliveira[2], Juliana Santana de Curcio[1], Paola Cristina Resende[3], Marilda Mendonça Siqueira[3], Alex Pauvolid-Corrêa[4], Gabriela Rodrigues Mendes Duarte[2], Elisângela de Paula Silveira-Lacerda[1*]

1- Laboratório de Genética Molecular e Citogenética, Departamento de Genética, Instituto de Ciências Biológicas I, Universidade Federal de Goiás, 74001-970, Goiânia, Goiás State, Brazil.

2- Instituto de Química – Universidade Federal de Goiás

3- Laboratory of Respiratory Viruses and Measles, Reference Laboratory for COVID-19 (WHO) of Oswaldo Cruz Foundation (Fiocruz), Rio de Janeiro, Brazil

4- Department of Veterinary Integrative Biosciences, Texas A&M University, College Station, Texas, United States of America

Supplementary Material

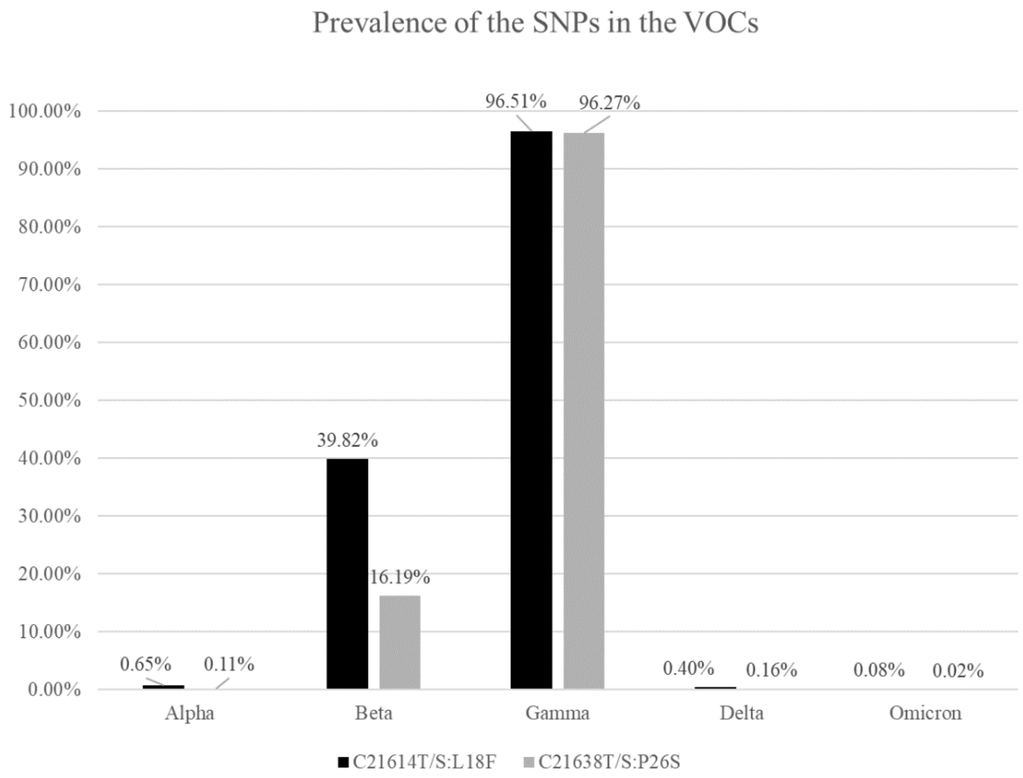

Figure S1. Prevalence of the mutations C21614T (also known as S:L18F) and C21638T (also known as S:P26S) in the five main variants of concern. These mutations are highly prevalent (>95%) only in the Gamma variant.

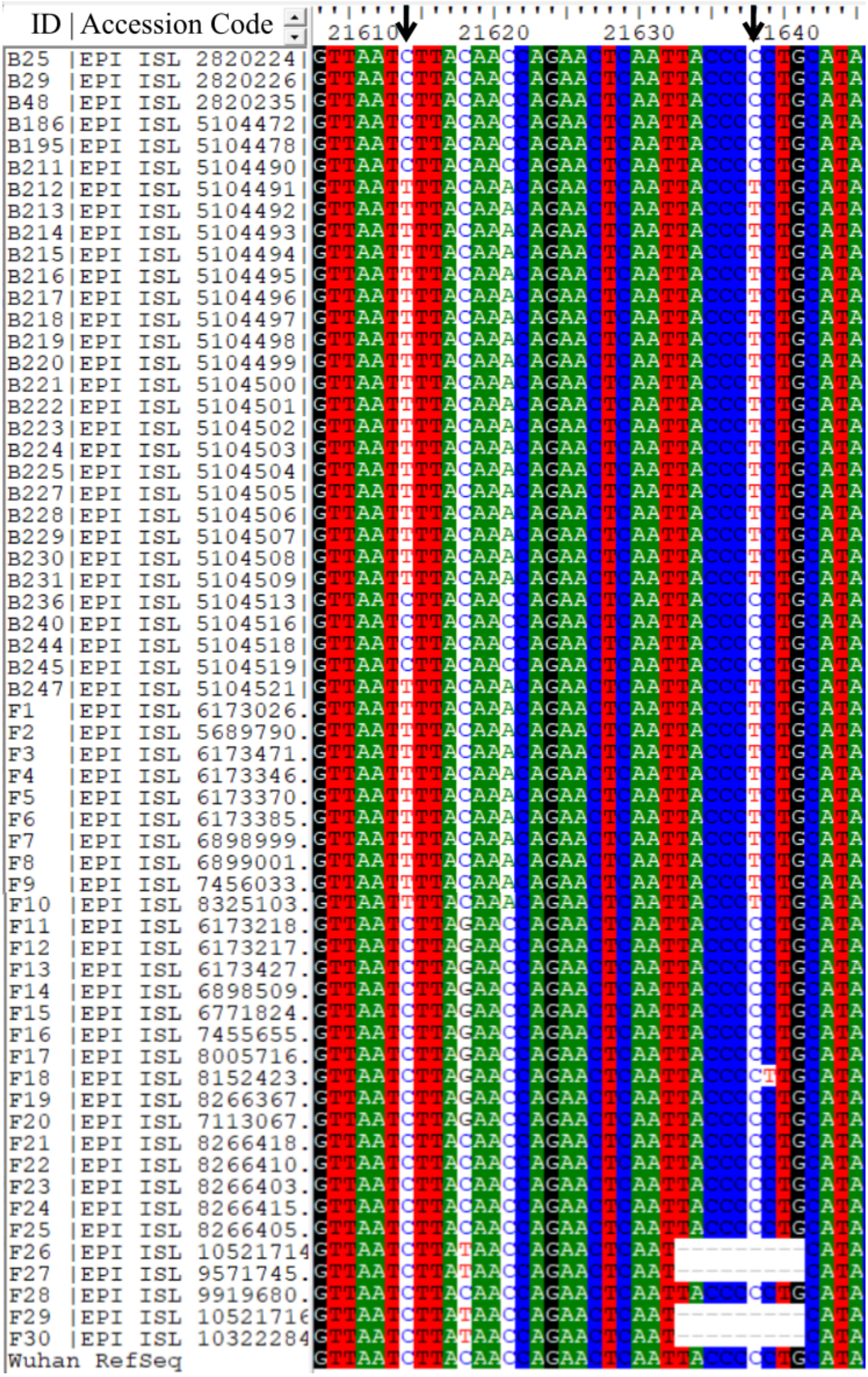

Figure S2. Aligned sequences of the samples included in this study. Samples number B212, B213, B214, B215, B216, B217, B218, B219, B220, B221, B222, B223, B224, B225, B227, B228, B229, B230, B231, B247, F1, F2, F3, F4, F5, F6, F7, F8, F9 and F10 harboured the mutations C21614T and C21638T. GISAID Accession Codes are presented in front of the

sample identification number. Black arrows indicate the position of the mutations in the sequences. For reference, sequences are compared to the Wuhan Reference Sequence (Accession Code on GenBank: NC_045512).

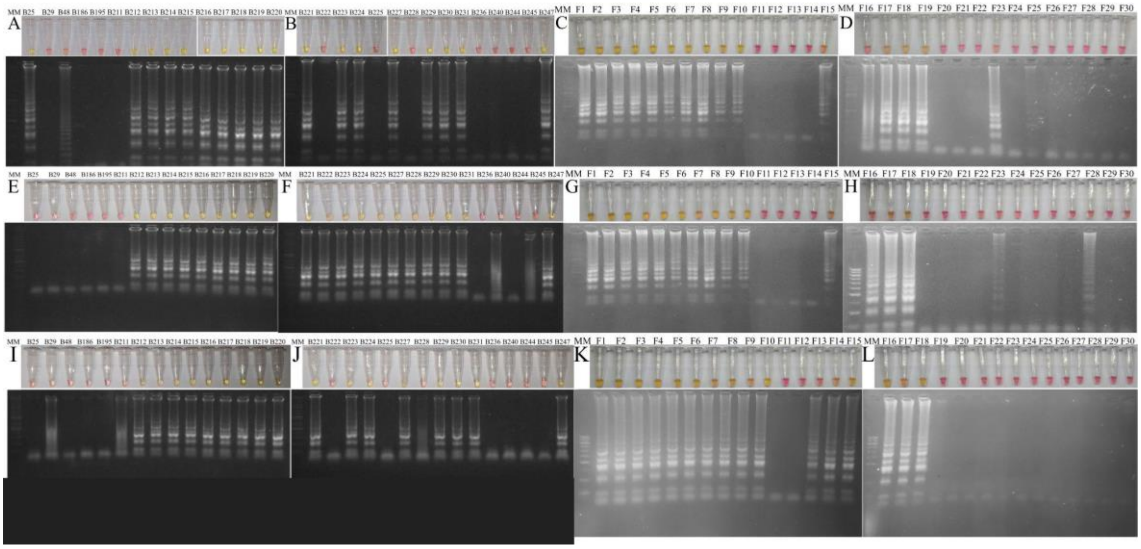

Figure S3. A, B, C and D. First replicate of the tested samples. The photos of the tubes were grouped together and aligned to their respective agarose gel wells for ease of visualisation. **E, F, G and H.** Second replicate of the tested samples. **I, J, K and L.** Third replicate of the tested samples. Numbers represent the number of the sample, and the colour of the tubes indicates whether there was amplification or not, yellow represents a positive result and pink represents a negative result. On the bottom of each row of tubes is the agarose gel as visualized in the photodocumentator. It is possible to visualize a distinct banding pattern on the agarose gel for sample code B48 in A, samples codes F16, F23 and F25 in D, samples B240 and B245 in F, F16, F23 and F28 in H, samples B29 and B211 in I, sample B228 in J and F13 in K, which are probably the result of spurious amplification. Even though these samples presented some banding patterns, as they did not show any significant color change they were considered negative. MM: Molecular Marker 100bp.

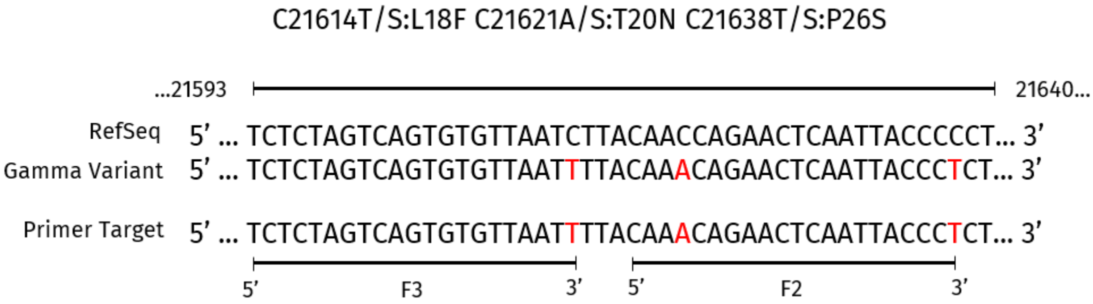

Figure S4. Primer targets and lineage-defining mutations in the Gamma variant. The primer targets the C21614T, and C21638T mutations common to the Gamma variant. Regions and nucleotides carrying the mutations are depicted in red. A line represents the region to which each primer anneals. Although the mutation C21621A is also present in primer F2, it was not considered for primer specificity as mutations in the 5' prime end have little to no effect on primer binding.

Table S1. Samples included in the analysis and their respective GISAID and/or GenBank accession codes.

Sample	GISAID Accession Code	GenBank Accession Code
B25	EPI_ISL_2820224	ON148300
B29	EPI_ISL_2820226	ON148301
B48	EPI_ISL_2820235	ON148302
B186	EPI_ISL_5104472	ON148303
B195	EPI_ISL_5104478	ON148304
B211	EPI_ISL_5104490	ON148305
B212	EPI_ISL_5104491	ON148306
B213	EPI_ISL_5104492	ON148307
B214	EPI_ISL_5104493	ON148308
B215	EPI_ISL_5104494	ON148309
B216	EPI_ISL_5104495	ON148310
B217	EPI_ISL_5104496	ON148311
B218	EPI_ISL_5104497	ON148312
B219	EPI_ISL_5104498	ON148313
B220	EPI_ISL_5104499	ON148314
B221	EPI_ISL_5104500	ON148315
B222	EPI_ISL_5104501	ON148316
B223	EPI_ISL_5104502	ON148317
B224	EPI_ISL_5104503	ON148318
B225	EPI_ISL_5104504	ON148319
B227	EPI_ISL_5104505	ON148320
B228	EPI_ISL_5104506	ON148321
B229	EPI_ISL_5104507	ON148322
B230	EPI_ISL_5104508	ON148323
B231	EPI_ISL_5104509	ON148324
B236	EPI_ISL_5104513	ON148325
B240	EPI_ISL_5104516	ON148326
B244	EPI_ISL_5104518	ON148327
B245	EPI_ISL_5104519	ON148328
B247	EPI_ISL_5104521	ON148329
F1	EPI_ISL_6173026	
F2	EPI_ISL_5689790	
F3	EPI_ISL_6173471	
F4	EPI_ISL_6173346	
F5	EPI_ISL_6173370	
F6	EPI_ISL_6173385	
F7	EPI_ISL_6898999	
F8	EPI_ISL_6899001	

F9	EPI_ISL_7456033
F10	EPI_ISL_8325103
F11	EPI_ISL_6173218
F12	EPI_ISL_6173217
F13	EPI_ISL_6173427
F14	EPI_ISL_6898509
F15	EPI_ISL_6771824
F16	EPI_ISL_6771824
F17	EPI_ISL_8005716
F18	EPI_ISL_8152423
F19	EPI_ISL_8266367
F20	EPI_ISL_8266367
F21	EPI_ISL_8266418
F22	EPI_ISL_8266410
F23	EPI_ISL_8266403
F24	EPI_ISL_8266415
F25	EPI_ISL_8266405
F26	EPI_ISL_10521714
F27	EPI_ISL_9571745
F28	EPI_ISL_9571745
F29	EPI_ISL_10521716
F30	EPI_ISL_10322284
	Controls:
Alpha	EPI_ISL_1402430
Gamma	EPI_ISL_1402431
B.1	EPI_ISL_414045
Zeta	EPI_ISL_2731468.2
